# Individual and population diversity of 20 representative olfactory receptor genes in pigs

**DOI:** 10.1038/s41598-023-45784-y

**Published:** 2023-10-31

**Authors:** Mingue Kang, Byeongyong Ahn, Seungyeon Youk, Hyoim Jeon, Nagasundarapandian Soundarajan, Eun-Seok Cho, Woncheoul Park, Chankyu Park

**Affiliations:** 1 Department of Stem Cell and Regenerative Biotechnology, Konkuk University, Seoul, 05029 Republic of Korea; 2 Rural Development Administration, National Institute of Animal Science, Wanju, 55365 Republic of Korea

**Keywords:** Evolution, Genetics, Molecular biology

## Abstract

Understanding the influence of genetic variations in olfactory receptor (OR) genes on the olfaction-influenced phenotypes such as behaviors, reproduction, and feeding is important in animal biology. However, our understanding of the complexity of the OR subgenome is limited. In this study, we analyzed 1120 typing results of 20 representative OR genes belonging to 13 OR families on 14 pig chromosomes from 56 individuals belonging to seven different breeds using a sequence-based OR typing method. We showed that the presence of copy number variations, conservation of locus-specific diversity, abundance of breed-specific alleles, presence of a loss-of-function allele, and low-level purifying selection in pig OR genes could be common characteristics of OR genes in mammals. The observed nucleotide sequence diversity of pig ORs was higher than that of dogs. To the best of our knowledge, this is the first report on the individual- or population-level characterization of a large number of OR family genes in livestock species.

## Introduction

Olfactory receptors (ORs) are members of the seven transmembrane G protein coupled receptor (GPCR) superfamily and are expressed mainly on olfactory neurons^1^. ORs bind to volatile odorants present in the air and transmit olfactory signals from bound odorants to the brain^2,3^. However, gene expression analysis of ORs in non-neuronal tissues, including testis, lung, intestine, skin, heart, and blood indicate their additional functions in modulating cell–cell recognition, cell migration, proliferation, pathfinding, and secretion^4–10^.

The sense of smell integrates a plurality of olfactory signals created by the binding of odorants to ORs; thus, a large degree of polymorphism is necessary to distinguish various olfactory cues by OR molecules^11,12^. OR genes constitute the largest gene family in mammalian genomes, and the size of the OR repertoire for intact OR genes in terrestrial tetrapods varies from < 400 in primates to 4267 in African elephants^13^. In addition, the percentage of OR pseudogenes among all OR genes in the genome have increased significantly in certain species favoring a reduction in the OR repertoire. For example, whales show a significant increase in OR pseudogenes compared to their terrestrial counterparts^14–16^.

OR genes are traditionally divided into two classes, class I and class II, based on amino acid sequence homology^17,18^. Class II OR genes have a much larger number of genes than class I genes and form clusters on multiple mammalian chromosomes. In contrast, class I OR genes commonly exist as a single cluster on chromosomes in mammalian species^19–22^. However, detailed characteristics of the functions and phylogenetic relationships of each class of ORs have been studied in a limited number of species^23–25^.

In addition, the allelic diversity of OR genes in an individual or population can influence their olfactory specificity and sensitivity by modulating the perception of odorants. A recent study showed that genetic variation in a single OR is frequently associated with changes in odorant perception in humans^26^. Another study showed that the allelic distribution of OR genes varies among different dog breeds^27–29^. However, the extent of allelic diversity of OR genes and the effects on phenotypic differences are not yet clearly understood due to the difficulty in identifying associations between genetic variations in a large number of OR genes and olfaction-related phenotypes. In addition, the large genetic variations in OR genes, including pseudogenization, nucleotide substitutions, indels, and copy number variations (CNVs), make it challenging to evaluate the genome-scale allelic diversity of OR genes in an individual genome.

No studies on allelic diversity and population variation of OR genes in livestock animals have been reported to date. Understanding the genetic variation in the OR repertoires of livestock species may reveal interesting aspects of ORs in animal behavior, reproduction, and feeding. For example, the stereotypical mating posture of an estrus female pig exposed to a compound in the saliva of boars is also mediated by the olfactory system^30^.

The OR repertoire of pigs (*Sus scrofa*) consists of 1301 OR genes, including 1113 functional and 188 pseudogenes^22,31^, which is much higher than the OR repertoire of cattle (970), which is another artiodactyl, and dogs (811), who are known for their excellent olfactory abilities^15,32^. Pigs are attractive animal models for studying olfaction due to their agricultural importance and strong reliance on their sense of smell in various behavioral contexts^33^. Approximately 200 to 300 breeds of pigs and wild boars exist worldwide^34,35^. Studies on OR diversity in diverse pig breeds provide important insights into how OR gene diversity is maintained and differentiated in populations.

A previous study showed that OR genes in the pig genome exist in 46 clusters scattered across 16 chromosomes and can be classified to 17 gene families^22^. In this study, we developed primer sets allowing the specific amplification of 20 pig OR genes covering 13 OR families on 14 pig chromosomes which include a large part of pig OR gene families. We compared the allelic diversity of each OR gene across 56 animals from seven pig breeds. Despite a limited population size for each breed, our results showed a significant difference in allelic constitution and diversity among different breeds.

## Results

### Successful development of a sequence-based typing (PCR-SBT) of 20 pig OR genes

To understand the genetic characteristics of OR genes in pigs, we selected 20 pig OR genes belonging to 13 OR families (1, 2, 5, 6, 8, 9, 10, 11, 13, 14, 51, 52, and 53) located on 14 chromosomes (chr1, 2, 4, 5, 6, 7, 9, 10, 12, 13, 14, 15, 18, and X) according to the OR classification in our previous study^22^ and the available sequence information in the pig genome assembly Sscrofa11.1 (NCBI; S1 Table). To construct a representative list of OR genes for the pig genome, we compared the previously identified OR genes based on the pig genome assembly Sscrofa10.2 from a prior study with those from the current assembly, Sscorfa11.1. The majority of identified OR genes were consistent between the two assemblies, with some minor discrepancies. Notably, the comparison revealed the presence of *sOR5P1* exclusively in Sscrofa10.2, while *sOR8F2P*, a pseudogene, initially located in the telomeric cluster of chr10 in Sscrofa10.2, had been relocated to unplaced scaffold NW_018085136.1 in Sscrofa11.1 (S1 Table).

To unbiasedly assess the genetic diversity of pig OR genes, we aimed to encompass sequences from at least one OR gene within each OR gene family. However, we were unable to include sequences of four OR families, 3, 4, 7, and 12, due to failure in locus-specific amplification. A pseudogene, *sOR8F2P,* was incorporated to address the influence of loss of function on the genetic diversity of OR genes. Consequently, a total of 20 OR genes representing 13 OR gene families across 14 pig chromosomes were selected, forming the basis for evaluating the allelic diversity of pig OR genes. Furthermore, an assessment of the expression of these selected OR genes was conducted utilizing publicly accessible data (NCBI Gene Expression Omnibus repository, accession number GSE197184; available at https://www.ncbi.nlm.nih.gov/geo/). The study revealed the expression of 18 out of the 20 chosen OR genes in the pig olfactory epithelium. Notably, *sOR5P1* and *sOR8F2P* were exceptions, as they were absent from the OR gene annotation of Sscrofa 11.1 (S2 Table).

Locus-specific primers were designed for each of the 20 OR genes (S3 Table), and PCR was conducted using the genomic DNA of 56 pigs from seven breeds (eight individuals each) including Berkshire (BER), Duroc (DUR), Korean Native (KNP), Landrace (LAN), National Institutes of Health miniature (NIH), Seoul National University miniature (SNU), and Yorkshire pigs (YOR) for each OR. PCR amplicons were obtained and sequenced to confirm target specificity. PCR primers were reiteratively designed to achieve locus-specific amplification of the target OR genes from all animals. Allelic determination of the 1120 typing results was conducted by blasting the sequencing results of amplicons to the OR reference sequences and pig reference genome. After cloning, a minimum of three colonies were independently analyzed to confirm a new allele for heterozygotes and to separate each allele. Finally, we obtained successful typing results for the 20 pig OR genes at the sequence level, and the sequences were submitted to GenBank (accession numbers OQ379512 – OQ379644) (S4 and S5 Tables).

### Confirmation of copy number variation for *OR53A1*

Our typing results showed that OR sequences from 18 out of 20 OR genes were mapped to a single chromosomal position, but *sOR6Y2* and *sOR53A1* were mapped to two different positions: *S. scrofa* chromosome (SSC) 14 90982296–90983234 (expected) and 91028428–91029366 (additional) and SSC9 3549170–3550117 (expected) and 3858315–3859262 (additional) bp regions, respectively (S3 Table, S1 Fig). This finding indicates the possible presence of CNV or gene duplication in the two OR genes in the pig genome. To experimentally validate the results, we designed additional primers specific to each of the differently mapped *sOR6Y2* and *sOR53A1* sequences based on identified sequence variations between the OR sequences mapped to two different regions and analyzed differences in amplification specificity in our sample panel (29 for *sOR53A1* and 20 for *sOR6Y2*; S2 Fig). For *sOR6Y2*, only the SSC14 90982296–90983234 bp (expected) region-specific sequences were amplified for all samples, but none of the animals possessed the 91028428–91029366 bp (additional) region-specific sequence. In contrast, 18 out of 29 animals (38%) showed a 3858315–3859262 bp region-specific additional sequence for *sOR53A1* (S2 Fig). Therefore, we experimentally confirmed the presence of CNV in *sOR53A1*. Subsequent RT-qPCR specific to all *sOR53A1*-like sequences residing at both SSC9 3549170–3550117 and 3858315–3859262 in our sample panel showed the presence of CNVs in SNU and LAN individuals (S6 Table, S2 Fig). The estimated copy number ranged from two to five. The increase in the *sOR53A1* copy number was associated with specific alleles, including sOR53A1*02 and *05 in SNU and sOR53A1*05 in LAN (S6 Table). We considered all *sOR53A1*-like sequences as allelic forms because we were unable to differentiate locus-specific differences among the *sOR53A1* amplicons.

### Allelic diversity of 20 pig OR genes and the presence of nonfunctional alleles

The typing results corresponding to the 20 pig OR coding sequences (CDS) ranging from 912 to 1038 bp (304–346 amino acids) in length from 56 animals belonging to seven pig breeds resulted in the identification of 351 single nucleotide polymorphisms (SNPs) (S7 Table). The number of SNPs for each OR gene ranged between 1 and 75, with a mean of 17.55. We assigned putative allele names for each OR allele in which two-digit numbers were given as allele names and separated with the “*” symbol after the locus names, as in sOR10L1*07, shown above. The allele with the highest frequency was assigned to *01, and additional numbers were assigned in the decreasing order of allele frequencies. Among these variations, a nucleotide deletion in the transmembrane 3 region of sOR10L1*07 causes premature termination in LAN pigs (S5 Table, S3 Fig). In addition, an SNP (T→G) at the first position of the stop codon of sOR13A6*03 resulted in disruption of the stop codon (S5 Table, S4 Fig). Notably, sOR13A6*03 was the major allele in LAN along with sOR13A6*01, with a frequency of 0.38 (S8 and S10 Tables).

**Figure 1:**
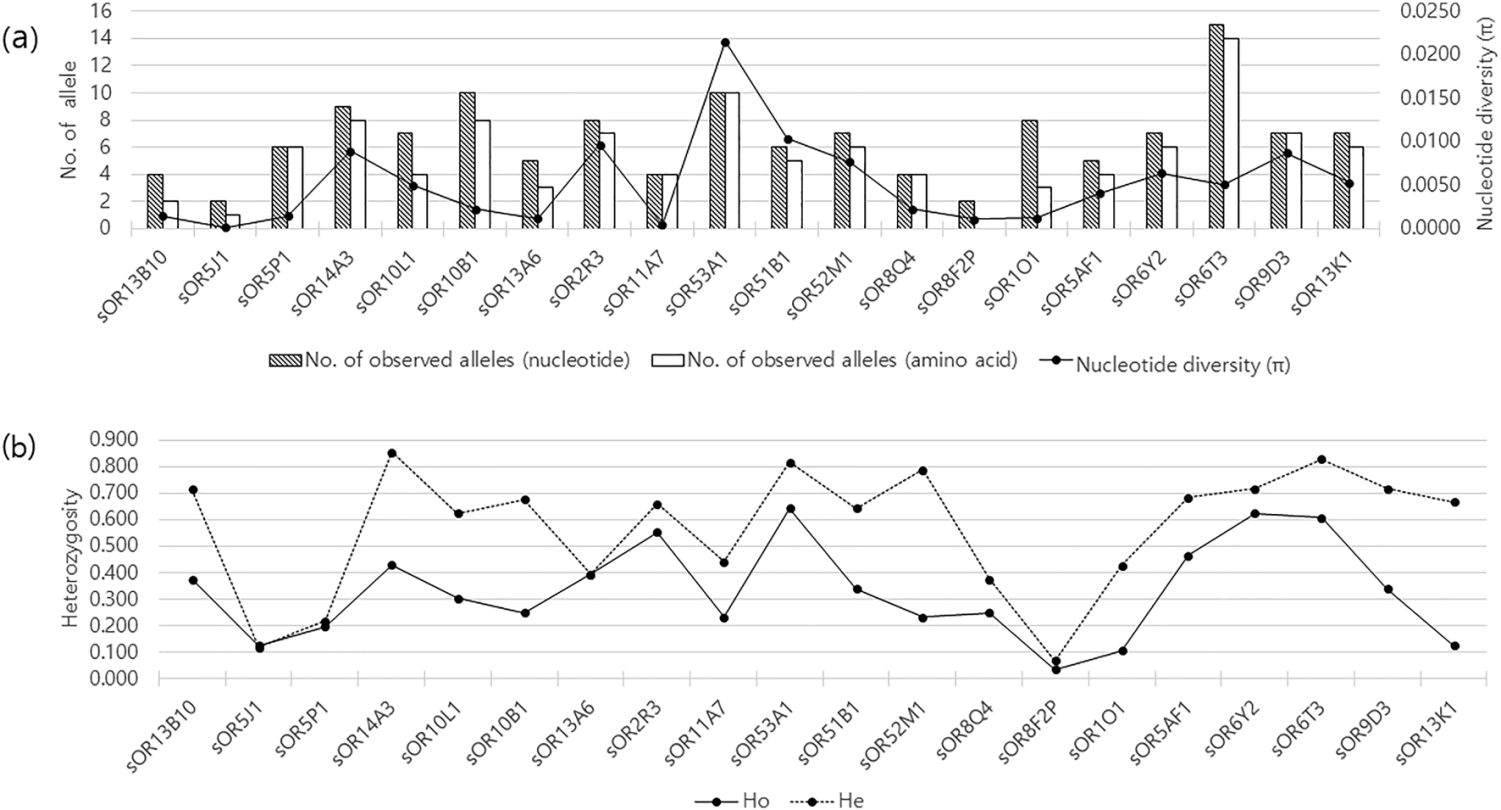
Allelic diversity of 20 pig olfactory receptor genes analyzed from seven pig breeds. (**a**) The mean number of alleles and mean nucleotide diversity (π) of 20 OR genes from the typing results of 56 pigs from seven breeds were plotted. The scale bars are indicated on the left and right, separately. (**b**) The mean heterozygosity (observed and expected) was plotted.

A significant difference was observed in the number of alleles for the OR genes, ranging from two (*sOR5J1* and *sOR8F2P*) to 15 (*sOR6T3*; Fig. 1). However, allelic diversity is not always proportional to the number of SNPs observed in each OR gene. For example, the number of alleles for *OR53A1* and *sOR6T3* was 10 and 15, respectively, whereas the corresponding number of SNPs were 75 and 28, respectively (S7 Table). This could be attributed to the abundant presence of rare mutations in an allele from the OR genes with a large number of alleles, such as *sOR6T3*.

The mean allele numbers (Na) of 20 OR genes at the nucleotide and amino acid sequence level were 6.65 and 5.68, respectively (Fig. 1, S7 Table), indicating a minimum level of reduction in allelic numbers in protein sequences. Most OR genes showed minimal differences (≤ 3) in allele numbers between nucleotide and protein sequences, except for *sOR1O1*, in which only three protein sequences were coded from eight alleles. In the case of *sOR1O1*, the ratio of nonsynonymous to synonymous SNPs (nd/sd) was low (0.167) compared to that of other ORs (S7 Table). The nd/sd ratio of the ORs ranged from 0 to 2.500, with a total mean of 1.014. The mean value of the nucleotide diversity (π) of the 20 pig OR genes was 0.0052, ranging from 0.0001 to 0.0216 (S7 Table). Positive correlations between π and the cluster size, and between π and the number of OR genes in the cluster were observed, with correlation coefficients I of 0.52 and 0.42, respectively (Table 1).

**Table 1:** Correlation between OR gene related genetic characteristics.

Parameter	Cluster size (bp)	No. of OR genes in a cluster	CDS length of OR genes (bp)
No. of OR genes in a cluster	0.95		
CDS length of OR genes (bp)	0.36	0.36	
Nucleotide diversity (π)	0.52	0.42	–0.20

Parameter values for the correlation analysis are described in S7 Table.

### Difference and similarity of pig OR genes among different breeds

Allelic diversity of 20 OR genes was compared among the seven pig breeds (Table 2). The average value of the Na of the 20 OR genes of each of the seven breeds was 2.607, ranging from a minimum of 1.750 in NIH to a maximum of 3.500 in LAN. The comparison among the breeds showed that the value was much higher in commercial breeds (mean of 2.810), including BER, DUR, LAN, YOR, and KNP, than in inbred miniature pigs (mean of 2.100), such as NIH and SNU. Moreover, we observed the presence of breed-specific alleles in 18 of the 20 OR loci, except for *sOR13B10* and *sOR5J1* (S10 Table). The largest number of breed-specific alleles (n = 3) was identified in *sOR6T3* of NIH. The mean value of breed level observed heterozygosity (Ho) from the typing results of 20 ORs for the seven pig breeds was estimated to be 0.331, ranging between 0.036 and 0.643 (Table 2 and S7 Table), showing significant differences in Ho among different breeds. LAN showed the highest values for Ho (0.469), with an Na of 3.500 and a mean breed-specific allele number (Np) of 0.700. NIH showed the lowest Ho (0.181) and was homozygous for 12 out of 20 OR genes (60%). For DUR, KNP, SNU, and YOR, allelic diversity was similar.

**Table 2:** Comparison of the mean allelic diversity of 20 OR genes among seven pig breeds.

	BER	DUR	KNP	LAN	NIH	SNU	YOR	Mean value
Na^a^	2.750	2.650	2.250	3.500	1.750	2.450	2.900	2.607
Ne^b^	1.859	1.953	1.696	2.210	1.411	1.823	1.893	1.835
I^c^	0.681	0.667	0.541	0.864	0.315	0.606	0.711	0.626
Np^d^	0.200	0.400	0.150	0.700	0.300	0.350	0.450	0.364
Ho^e^	0.344	0.338	0.281	0.469	0.181	0.338	0.369	0.331
He^f^	0.416	0.407	0.348	0.492	0.200	0.378	0.422	0.380

^a^number of alleles, ^b^number of effective alleles, ^c^Shannon’s information index, ^d^number of private alleles, ^e^observed heterozygosity, ^f^expected heterozygosity.

The extent of locus-specific OR diversity was consistent across different breeds. For example, the allelic diversity of *sOR5J1* was the lowest across all seven breeds among functional ORs, and similarly, that of *sOR53A1* was the highest among all breeds typed (Fig. 1, S8 Table). *sOR5J1* and *sOR5P1* were homozygous in most animals across all seven breeds, and sOR5J1 was fixed to a single allele at BER, DUR, KNP, NIH, and SNU (S8 and S11 Tables). This finding could suggest the characteristics of each OR gene regarding functional restriction or diversity in the detection of various odorants. Contrary to expectations, *sOR8F2P*, a pseudogene, showed fixation of an allele in most breeds, except SNU. In addition, we observed nine alleles (sOR5P1*01, sOR5J1*01, sOR13A6*01, sOR1A7*01, sOR53A1*02, sOR8Q4*01, sOR8F2P*01, sOR5AF1*01, and sOR2R3*01) common across all seven breeds (S8 and S9 Tables). The frequency of these common alleles was relatively high in all analyzed breeds, with frequencies of 0.88 (sOR5P1*01) and 0.54 (sOR2R3*01) in the breed combined analysis (S8 Table). In addition, sOR10L1*07, a non-functional allele identified in this study, was observed in a LAN individual in the form of a heterozygote (S3 and S8 Tables).

**Figure 2:**
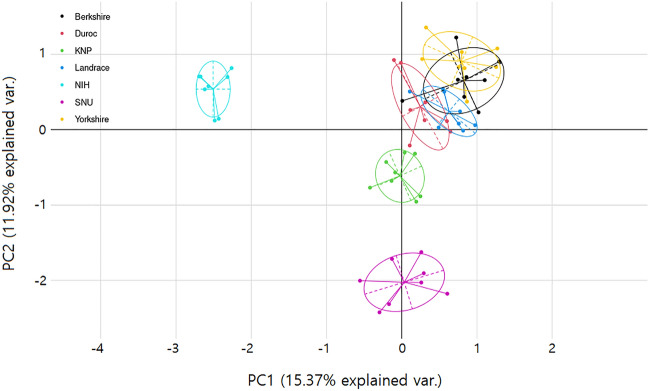
The results of principle component analysis among different pig breeds from the genotype information of 20 OR genes. The results of principle component analysis using principal component (PC) 1 and 2 are shown. Each individual is represented by a dot, and different breeds are indicated by different colors, as shown on the upper left of the plot.

A principal component analysis (PCA) was conducted using the typing results of 20 OR genes from all seven breeds (Fig. 2). The results using principal components 1 and 2 showed the clustering of the European breeds, including BER, DUR, YOR, and LAN, and the formation of separate clusters for NIH and SNU, respectively. Moreover, KNP was somewhat separated from the cluster of European breeds but was closer to the European breeds than to the miniature pigs.

### Higher inter-locus differences of class I OR over class II OR genes

**Figure 3:**
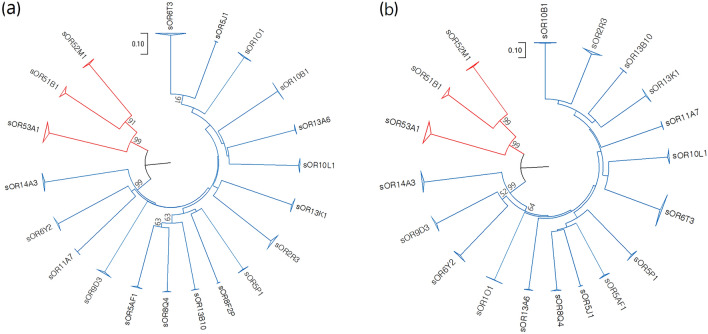
Phylogenetic relationships of 20 pig OR genes. UPGMA trees were plotted based on nucleotide (**a**) and protein sequences (**b**), respectively. Alleles were clustered together for all 20 OR genes and, therefore, alleles of the same OR genes were collapsed and indicated by triangles at the end of the branch. Branches of Class I and II OR genes are indicated in red and blue, respectively. Bootstrap values less than 50% are not shown in the plots. The genetic distance is scaled at the upper center.

To understand the genetic relationships of the 20 OR genes analyzed in our study, a unweighted pair group method with arithmetic mean (UPGMA) tree was constructed from the typing results of the 20 OR genes using nucleotide and protein sequences (Fig. 3). The combined analysis of three class I genes *(sOR53A1, sOR51B1*, and *sOR52M1*) located in a cluster on SSC9 and 17 class II OR genes (*sOR13B10, sOR5J1, sOR5P1, sOR14A3, sOR10L1, sOR10B1, sOR13A6, sOR2R3, sOR11A7, sOR8Q4, sOR8F2P, sOR1O1, sOR5AF1, sOR6Y2, sOR6T3, sOR9K3*, and *sOR13K1*) located on 14 different chromosomes formed two separate clusters. The OR clusters formed subclusters within each cluster, indicating that the 20 OR genes selected in this study represent a large genetic diversity of pig OR genes. The higher bootstrapping values (> 90%) of class I OR subfamilies relative to those of class II OR subfamilies in clustering analysis indicate clear inter-locus differences among class I OR genes as compared to class II OR genes. The trees constructed using nucleotide and amino acid sequences showed consistent results, but the clustering of OR genes in the branches with low bootstrapping values was less conclusive.

In addition, the mean nucleotide diversity of class I OR genes (π_mean_ = 0.0132) was significantly higher (paired sample *t*-test, *P* = 0.000366) than that of class II OR genes (π_mean_ = 0.0038; S7 Table). This finding may indicate an evolutionary effect against the duplication of class I OR genes in mammals to maintain a relatively lower number of class I OR genes than class II ORs by limiting the functional redundancy of class I OR repertoires. However, when comparing the mean Ka/Ks ratio between class I and II OR genes with more than 10 SNPs (*sOR5P1, sOR14A3, sOR10L1, sOR10B1, sOR2R3, sOR1O1, sOR5AF1, sOR6Y2, sOR6T3, sOR9D3, sOR13K1, sOR53A1, sOR51B1,* and *sOR52M1*), most of them were under weak purifying selection and no significant difference was observed in the ratio between the two (Ka/Ks _class I_ = 0.307, Ka/Ks _class II_ = 0.316, Student’s *t*-test, *P* = 0.935; S7 Table). This result could be attributed to the relatively recent gene expansion in Class II OR genes compared to Class I OR genes.

### Extent of genetic variation among different structural domains of pig OR genes

Pig OR genes consist of 15 structural domains, including N-terminal (Nterm), C-terminal (Cterm), seven transmembrane (TM1–7), three extracellular (EC1–3), and three intracellular domains (IC1–3)^3,36^. The domain structures of 133 different sequences from our typing results of 1120 OR amplicons were determined based on conserved sequences for each motif, and intra-locus variations were analyzed for each domain. Values of the number of variable sites, pairwise differences, and Ka/Ks ratio were estimated for each domain of the 20 OR genes (Table 3, S12 Table, S5 Fig). Although the number of variable sites were somewhat different from 0 to 18 for the 15 OR domains, the normalized values for the length (in bp) of each domain were not significantly different. The percentage of variable sites for each domain ranged from 1 to 3% of total sites, with an average of 1.93%, whereas TM5 and TM6 were slightly higher (2.85 and 2.62%, respectively) than IC1 and TM3 (1.17 and 1.39%, respectively). The Ka/Ks ratio of each structural domain showed that all OR domains were under purifying selection, with values ranging from 0.020 to 0.826 in pigs (Table 3).

**Table 3:** Comparison of the genetic diversity of different OR structural domains from 20 pig OR genes.

Domain^a^	Mean value from 20 OR genes analyzed in this study						
	Length (A) (bp)	No. of variable sites (B)	(B)/(A) ratio (%)	π	nd^b^	sd^c^	Ka/Ks^d^
Nterm	76.6	1.16	1.51	0.0065	0.84	0.32	0.772
TM1	84.0	1.53	1.82	0.0074	0.95	0.58	0.826
IC1	18.0	0.21	1.17	0.0049	0.11	0.11	0.630
TM2	87.0	1.74	2.00	0.0088	0.89	0.84	0.306
EC1	21.2	0.37	1.74	0.0066	0.21	0.16	0.267
TM3	102.0	1.42	1.39	0.0056	0.79	0.63	0.383
IC2	30.0	0.47	1.58	0.0064	0.26	0.21	0.512
TM4	63.0	1.16	1.84	0.0074	0.74	0.42	0.635
EC2	108.9	2.11	1.93	0.0086	0.95	1.26	0.216
TM5	88.7	2.53	2.85	0.0128	1.42	1.11	0.449
IC3	23.8	0.37	1.55	0.0056	0.05	0.32	0.031
TM6	100.4	2.63	2.62	0.0103	1.05	1.58	0.219
EC3	21.6	0.53	2.43	0.0104	0.05	0.47	0.020
TM7	63.0	1.16	1.84	0.0054	0.21	0.95	0.068
Cterm	60.6	0.95	1.56	0.0066	0.42	0.53	0.182
Mean	63.3	1.22	1.93	0.0075	0.60	0.63	0.368

^a^Nterm, N-terminal; TM, transmembrane; IC, intracellular; EC, extracellular; Cterm, C-terminal; ^b^the mean number of non-synonymous sites; ^c^the mean number of synonymous sites; ^d^Ka, number of nonsynonymous substitutions per nonsynonymous site; Ks, number of synonymous substitutions per synonymous site.

## Discussion

Olfaction is critical for the survival of several animal species. OR genes are highly expanded in mammals, who largely rely on smell for finding food, recognizing danger, and reproducing. The genetic variations in OR genes are likely to be associated with functional differences in olfaction, together with anatomical differences in the olfactory organs. Therefore, understanding the genetic differences in OR genes should allow for the prediction of the diversity and sensitivity of olfaction capacity in animal species. However, target-specific typing of OR genes in mammals is challenging because of the extremely large number of paralogs in their genomes and genetic variations^13,15,19,20,22^. Thus, systematic analyses of the genetic diversity of mammalian OR genes at the population level have been limited to a few studies^28,37,38^.

The number of OR genes varies greatly among species and reflects their dependency on the olfaction of each species. The analysis of OR subgenomes in mammalian species showed that pigs are animals with a large number (~ 1110) of functional OR genes^13,22,25^. Previous studies suggested that ORs with more than 60% identity in protein sequence may recognize odorants with related structures^11,39^. However, the presence of a large number of OR genes with > 60% of protein sequence similarity or subfamilies in the mammalian genomes indicate functional differences among closely related sequences. Nguyen et al.^22^ classified 349 OR subfamilies belonging to three class I and 14 class II OR families in the pig genome. In addition, allelic diversity can contribute to the functional diversity of a gene.

In this study, we analyzed the genetic variations of 20 annotated pig OR genes belonging to 13 OR families using locus-specific PCR-SBT typing to maximize the diversity of selected OR genes and presented the allelic diversity of OR genes in pigs. Although the number of OR genes analyzed were limited, we covered OR genes from 13 of the 17 families in the pig genome. Our results showed that pig OR genes have similar characteristics in genetic diversity to those of humans and dogs^27,28,40^ in the polymorphic spectrum of OR genes consisting of both weakly (mean π < 0.0052; i.e., *sOR13B10, sOR5J1, sOR5P1, sOR10L1, sOR10B1, sOR13A6, sOR11A7, sOR8Q4, sOR8F2P, sOR1O1, sOR5AF1, sOR6T3*) and highly polymorphic genes (mean π > 0.0052; i.e., *sOR53A1, sOR51B1, sOR52M1, sOR14A3, sOR2R3, sOR6Y2, sOR9D3, sOR13K1*; S7 Table). Compared to humans and dogs, pigs have a higher allelic diversity in OR genes belonging to large clusters than to small (Table 1), presence of breed-specific alleles (S10 Table), weak selection (S7 Table), and higher allelic diversity in class I over class II OR genes (S7 Table). Moreover, we observed significant differences in the allelic constitution of OR genes across different pig breeds, but the availability of data from other species is limited.

The nucleotide sequence diversity of ORs in pigs (mean π = 0.0052) observed in this study was significantly higher (Z-test, P = 0.00107) than that reported for dogs (π = 0.0018)^27,28^, consistent with the fact that the size of the pig OR repertoire is larger than that of dogs^21,22,32^, showing a positive correlation between the extent of gene expansion and nucleotide sequence variations in OR genes, which reflects inter-locus and intra-locus diversity, respectively. The number of OR genes in a given species can be positively correlated with the number of odorants that can be discriminated by the species. However, sensitivity to a specific odorant may be determined by the expression levels of OR genes and anatomical characteristics of the olfactory epithelium. Although speculative, the discrimination spectrum and specificity of the OR system to various odorants could be better in pigs than in dogs, considering the revealed genetic diversity of the OR genes of the two species.

The positive correlation between the extent of gene expansion and nucleotide sequence diversity of OR genes is interesting, suggesting the importance of olfaction in animal survival although the dependency varies on species. Animals with larger sizes of OR repertoire may also maintain higher allelic diversity than those with smaller OR repertoire. The higher level of inbreeding for domestic dog breeds than for pigs may also contributed to the reduced overall diversity of OR genes in domestic dogs^41,42^.

OR molecules consist of 15 structural domains, and the extent of genetic variation and conservation of each domain may vary. In previous studies, multiple alignments of numerous mammalian ORs showed that the extent of conservation was relatively low between TM3 and TM6^43^. In this study, we observed higher degrees of genetic variation in TM5 (π = 0.0128), TM6 (π = 0.0103), and EC3 (π = 0.0104) domains than in others (mean π = 0.0075) for pig ORs (Table 3). However, the mean Ka/Ks ratio of EC3 (0.020) was lower than that of TM5 (0.449) and TM6 (0.219), indicating a difference in domain diversification depending on the selective advantages of the region in olfaction.

Major histocompatibility complex (MHC) and OR are similar in that they recognize and differentiate a large number of ligands and have evolved to create high genetic diversity through gene duplication and allelic diversity. Therefore, the genetic characteristics of the two protein families are similar. For swine leukocyte antigen (*SLA*) genes, which is the MHC genes of pig, it has been well documented that the presence of an extremely large number of alleles, a large difference in allelic constitution among different breeds, and breed-specific alleles^44–49^ are most likely to result from independent breeding histories for different breeds. In addition, the characteristics of pig OR genes analyzed in this current study showed similarities to those of the *SLA* genes described above. Moreover, the observation of a larger number of OR alleles in LAN and YOR breeds than in other breeds is consistent with the results for *SLA* class I genes^45^. LAN and YOR are well-recognized as preferable maternal lines in pig breeding.

Conservation of the extent of the genetic diversity of individual OR genes across different pig breeds is interesting. This could suggest the characteristics of each OR gene regarding functional restriction or diversity in the detection of various odorants. The locus-specific diversity of individual OR genes could indicate either differences in the age of the locus after duplication or the permissibility of genetic changes among OR genes. A positive correlation between the genetic diversity of individual OR genes and the cluster size of OR genes suggests an association between gene duplication and the allelic diversity of OR genes, consistent with a previous study on dog ORs^27^.

Notably, only two alleles were observed for *sOR8F2P*, a pseudogene, across most breeds, despite the loss of selection pressure on pseudogenes^50–52^. In addition, we observed limited allelic diversity among a few OR genes, including *sOR5J1, sOR5P1, sOR11A7, sOR13B10,* and *sOR8Q4* (S7 Table). In contrast, larger genetic diversity was observed for *sOR14A3, sOR10B1, sOR2R3, sOR53A1, sOR6Y2,* and *sOR6T3* than for other ORs. The allelic diversity of individual OR genes may be related to the characteristics of the target odorants binding to individual OR molecules. In addition, we observed the presence of conserved alleles with a high frequency (> 0.5) in a few OR genes across all seven pig breeds (S13 Table), which may indicate the functional importance of these alleles.

The mean nucleotide diversity (π_pig_ = 0.0052) calculated from the 20 pig OR genes in this study was much higher than that of other species, including humans (π_human_ = 0.0008,^40^) and dogs (π_dog_ = 0.0018,^27,28^), suggesting accumulation of a larger number of genetic changes in pigs than in the other two species (approximately three times higher than in dogs). Therefore, the OR system of pigs has evolved to have large genetic diversity, together with a large number of functional OR genes and less pseudogenization than in humans or dogs.

The large number of OR genes in the genome and the structural characteristics of OR genes as a single exon gene may expose OR genes to continuous genetic changes. Thus, a large number of functional OR genes, as many as 400–2000 in mammals, has been dictated by continuous change in the OR repertoire, varying with the dependence on olfaction by individual species. Moreover, the size of the functional OR repertoire can rapidly decrease once the dependence on olfaction is weakened, as in marine mammals^16,25,53^.

Analysis of evolutionary dynamics from the 20 pig OR genes showed the presence of weak purifying selection consistent with that of previous studies^13,25,54^. However, our results indicated no clear difference in selection pressure between class I and class II OR genes (S7 Table), which differs from the results of a few previous studies^13,25,54^. This difference could be attributed to the differences in the characteristics of the datasets among the studies. Our analysis was based on the genetic variations of each OR locus separately, rather than using the combined genetic variations of OR orthologs in previous studies.

In conclusion, we presented the results of the population diversity of 20 representative pig OR genes belonging to 13 OR families. Genetic diversity varies among OR genes and depends on the breeding structure. The large number of OR genes in pigs indicates a large dependence on olfaction for their survival. Although this study was limited by the number of OR genes and individuals analyzed for each breed, we believe that the genetic characteristics of the remaining OR genes in the pig genome are likely to be consistent with the results presented here. Identification of the ligands for major OR alleles that are common across different breeds and for highly polymorphic OR genes may help clarify the characteristics of functionally critical ORs for pigs. Analyses of the genetic diversity of the complete OR repertoire of an individual and comparisons among different individuals, breeds, or other Suidae animals are challenging, considering the genetic complexities of OR genes described in this study. However, our study is the first step toward achieving these objectives.

## Methods

### Animals and tissues

Tissue samples of seven pig breeds, BER, DUR, LAN, YOR, KNP, NIH, and SNU, were collected and stored at -80 °C in previous studies^45,55^ and used to prepare genomic DNA for this study. Eight individuals from each breed were randomly selected. All experiments were approved and performed in accordance with the guidelines and regulations set by the Institute of Animal Care and Use Committee and Center for Research Ethics of Konkuk University.

### Preparation of genomic DNA

Genomic DNA (gDNA) was extracted from each tissue (0.5 g) using a standard protocol^56^. Tissues were briefly incubated with 700 µL lysis buffer (50 mM Tris pH 8.0, 0.1 M EDTA pH 8.0, 0.5% (w/v) sodium dodecyl sulfate, 20 μg/mL DNase-free pancreatic RNase) and 20 µL of proteinase K (20 mg/mL) at 50 °C overnight. After incubation, gDNA was isolated using phenol:chloroform:isoamyl alcohol buffer (pH 8.0) and subsequently precipitated and purified using ethanol precipitation methods .

### Primer design

Primers to specifically amplify each of 20 OR genes were designed using NCBI Primer-BLAST (https://www.ncbi.nlm.nih.gov/tools/primer-blast/). The 500-bp upstream and downstream regions of the selected OR genes, based on the pig genome assembly (Sscrofa 11.1), were used as queries, and the primer size was set to Min: 20, Opt: 23, Max: 25, expecting amplicons 1000–2000 bp in size. Off-target or multiple-target amplifications from the designed primers were tested using BLAST analysis.

### Polymerase chain reaction and sequencing

A single OR gene-specific PCR in a 10 µL reaction volume was performed for 20 OR genes using an ABI9700 thermocycler (Applied Biosystems, Foster City, CA). The reaction mixture consisted of 25 ng gDNA, 1 µM of each primer (S3 Table), 250 µM dNTP, 1U Supertherm™ Taq DNA polymerase (JMR Holdings, Kent, UK), and 10 × reaction buffer with 15 mM MgCl_2_ (JMR Holdings, Kent, UK). The PCR profile was 94 °C for 3 min of pre-denaturation, 30 cycles of 94 °C for 30 s, a specific annealing temperature for each primer set for 45 s, and elongation at 72 °C for 90 s, followed by a final elongation at 72 °C for 10 min. Amplification products were electrophoresed on 1% agarose gel to confirm target amplification. To sequence OR amplicons, sequencing primers for each OR gene were designed in both the forward and reverse directions using the Primer Designer of CLC Main Workbench 7.8.1 software (CLC bio, Aarhus, Denmark). To remove primer dimers and unincorporated dNTPs, 5 μL of PCR products were mixed with 0.25 U of shrimp alkaline phosphatase (USB Corporation, Cleveland, OH), 15 U of exonuclease I (Fermentas, Massachusetts, USA), and incubated at 37 ℃ for 30 min. The sequencing reaction was performed using the Applied Biosystems BigDye® Terminator v3.1 Cycle Sequencing Kit (Applied Biosystems, Massachusetts, USA) with 2 pmol of a sequencing primer (S3 Table) under the cyclic conditions of pre-denaturation at 96 °C for 1 min, 25 cycles of 96 °C for 10 s, 50 °C for 5 s, and 60 °C for 4 min. The reaction products were purified using ethanol precipitation, resuspended in 10 μL of Hi-Di™ Formamide (Applied Biosystems, Massachusetts, USA), and analyzed on an ABI3730 DNA Analyzer (Applied Biosystems).

### Allelic differentiation

The sequence information and annotations of pig OR genes reported by Nguyen et al.^22^ were used as a reference for the analysis of allelic variations of 20 selected pig OR genes. Nucleotide variations, including SNPs and indels, were identified by aligning the sequencing results of the OR genes to the reference sequences using CLC Main Workbench 7.8.1 software (CLC bio, Aarhus, Denmark). Alleles that existed as homozygotes were designated as novel alleles without any further analysis. Alleles that existed as heterozygotes were separated into individual alleles through TA cloning using pGEM®-T Easy Vector Systems (Promega, Wisconsin, USA), and the ligation products were transformed into DH10B competent cells (Thermo Fisher Scientific, Massachusetts, USA) using electro-transformation. Target inserts were confirmed by colony PCR using T7 and SP6 universal primers, 10 µL of colony PCR mixture containing a piece of a single bacterial colony as a DNA template, 1 µM of PCR primers, 250 µM dNTP, 1U of Supertherm™ Taq DNA polymerase (JMR Holdings, Kent, UK), and 10X reaction buffer with 15 mM MgCl_2_ (JMR Holdings, Kent, UK). The thermal profile for the colony PCR consisted of 5 min of pre-denaturation at 94 °C, 30 cycles of denaturation at 94 °C for 30 s, primer annealing at 50 °C for 30 s, and elongation at 72 °C for 60 s, followed by a final elongation at 72 °C for 7 min. PCR amplicons were sequenced using primers specific to each OR gene, as described above. At least eight independent clones were analyzed for allelic determination.

### Real-time quantitative PCR (qPCR)

Real-time quantitative PCR (qPCR) was performed in a 10 μL reaction containing 2X SYBR® Green Master Mix (Bio-Rad, California, USA), 1 µM of each primer (sOR53A1_qPCRF and sOR53A1_PCRR (S3 Table) and GCG forward and reverse^57^, respectively), and 25 ng of gDNA. PCR was run using the CFX Connect Real-Time Thermal Cycler System (Bio-Rad) under the following conditions: pre-denaturation at 94 °C for 3 min, 40 cycles of denaturation at 94 °C for 30 s, annealing at 65 °C for 10 s, and elongation at 72 °C for 15 s. The glucagon gene (*GCG*) was selected as a single-copy control gene for quantification of *sOR53A1*^58,59^. The efficiency of *sOR53A1* and *GCG* amplification was measured by plotting qPCR amplification standard curves three times using gradients of 125, 25, 5, and 1 ng of gDNA from an individual (sample ID: KNP3, a single copy homozygote, S6 Fig). The gene copy number of *sOR53A1* was estimated using the 2^-ΔΔCT^ relative quantification method^60^.

### Calculation of genetic diversity

Genetic diversity indices, including allele frequencies, expected (He) and observed heterozygosity (Ho), mean Ho and He, mean number of alleles (Na), mean effective allele number (Ne), mean number of private alleles (Np), and mean Shannon's information index (I), were calculated using GenAlEx 6.5^61^. The nucleotide diversity (π) was calculated using Arlequin 3.5.2.2^62^.

### Principle component analysis (PCA)

A dataset for PCA was prepared using the genotyping information of 20 OR genes from 56 individuals of seven pig breeds. This information was saved in the genind class of the adegenet R package (version 2.1.6). PCA was conducted using the dudi.pca function of the ade4 version 1.7.19 package in R version 4.1.2^63^.

### Phylogeny analysis

Nucleotide and amino acid sequence alignments were prepared using CLC Main workbench 7.8.1 (CLC bio, Aarhus, Denmark) with a gap open cost of 10.0 and gap extension cost of 1.0. The UPGMA algorithm was used to create a phylogenetic tree with 5000 bootstrap replicates, and the distance was estimated using the Jukes-Cantor method. The tree was saved in Newick format and visualized using MEGA version 11.0.10^64^.

### Evolutionary analysis

The mean ratio of the number of nonsynonymous substitutions per nonsynonymous site (Ka) to the number of synonymous substitutions per synonymous site (Ks; Ka/Ks ratio) for each OR gene was calculated as the average value of the Ka/Ks ratios for each allele for each OR gene. DnaSP v6.12.03^65^ was used to calculate the Ka/Ks ratio of each gene.

### Supplementary Information

Supplementary Figures.

Supplementary Tables.

## Data Availability

A total of 133 sequences corresponding to the alleles of 20 olfactory receptor genes analyzed in this study were submitted to NCBI GenBank (www.ncbi.nlm.nih.gov/genbank/) under accession numbers listed in S5 Table (OQ379512- OQ379644). All other information can be found in the text and supporting information.

## References

[1] Buck L, Axel R (1991). A novel multigene family may encode odorant receptors: A molecular basis for odor recognition. Cell.

[2] Firestein S (2001). How the olfactory system makes sense of scents. Nature.

[3] Gaillard I, Rouquier S, Giorgi D (2004). Olfactory receptors. Cell. Mol. Life Sci..

[4] Aisenberg WH (2016). Defining an olfactory receptor function in airway smooth muscle cells. Sci Rep.

[5] Braun T, Voland P, Kunz L, Prinz C, Gratzl M (2007). Enterochromaffin cells of the human gut: Sensors for spices and odorants. Gastroenterology.

[6] Busse D (2014). A synthetic sandalwood odorant induces wound-healing processes in human keratinocytes via the olfactory receptor OR2AT4. J. Investig. Dermatol..

[7] Kalbe B (2017). Helional-induced activation of human olfactory receptor 2J3 promotes apoptosis and inhibits proliferation in a non-small-cell lung cancer cell line. Eur. J. Cell Biol..

[8] Neuhaus EM (2009). Activation of an olfactory receptor inhibits proliferation of prostate cancer cells. J. Biol. Chem..

[9] Spehr M (2003). Identification of a testicular odorant receptor mediating human sperm chemotaxis. Science.

[10] Tsai T (2017). Two olfactory receptors-OR2A4/7 and OR51B5-differentially affect epidermal proliferation and differentiation. Exp. Dermatol..

[11] Malnic B, Hirono J, Sato T, Buck LB (1999). Combinatorial receptor codes for odors. Cell.

[12] Hallem EA, Carlson JR (2006). Coding of odors by a receptor repertoire. Cell.

[13] Niimura Y, Matsui A, Touhara K (2014). Extreme expansion of the olfactory receptor gene repertoire in African elephants and evolutionary dynamics of orthologous gene groups in 13 placental mammals. Genome Res..

[14] Kishida T, Kubota S, Shirayama Y, Fukami H (2007). The olfactory receptor gene repertoires in secondary-adapted marine vertebrates: Evidence for reduction of the functional proportions in cetaceans. Biol. Lett..

[15] Lee K (2013). Analysis of cattle olfactory subgenome: The first detail study on the characteristics of the complete olfactory receptor repertoire of a ruminant. BMC Genomics.

[16] Yim HS (2014). Minke whale genome and aquatic adaptation in cetaceans. Nat. Genet..

[17] Freitag J, Krieger J, Strotmann J, Breer H (1995). Two classes of olfactory receptors in *Xenopus laevis*. Neuron.

[18] Glusman G (2000). The olfactory receptor gene superfamily: Data mining, classification, and nomenclature. Mamm. Genome.

[19] Glusman G, Yanai I, Rubin I, Lancet D (2001). The complete human olfactory subgenome. Genome Res..

[20] Zhang X, Firestein S (2002). The olfactory receptor gene superfamily of the mouse. Nat. Neurosci..

[21] Quignon P (2005). The dog and rat olfactory receptor repertoires. Genome Biol..

[22] Nguyen DT (2012). The complete swine olfactory subgenome: Expansion of the olfactory gene repertoire in the pig genome. BMC Genomics.

[23] Niimura Y, Nei M (2005). Evolutionary dynamics of olfactory receptor genes in fishes and tetrapods. Proc. Natl. Acad. Sci. U. S. A..

[24] Niimura Y (2009). On the origin and evolution of vertebrate olfactory receptor genes: Comparative genome analysis among 23 chordate species. Genome Biol. Evol..

[25] Liu A (2019). Convergent degeneration of olfactory receptor gene repertoires in marine mammals. BMC Genomics.

[26] Trimmer C (2019). Genetic variation across the human olfactory receptor repertoire alters odor perception. Proc. Natl. Acad. Sci. U. S. A..

[27] Robin S (2009). Genetic diversity of canine olfactory receptors. BMC Genomics.

[28] Chen R, Irwin DM, Zhang YP (2012). Differences in selection drive olfactory receptor genes in different directions in dogs and wolf. Mol. Biol. Evol..

[29] Lesniak A (2008). Canine olfactory receptor gene polymorphism and its relation to odor detection performance by sniffer dogs. J. Hered..

[30] Dorries KM, Adkins-Regan E, Halpern BP (1997). Sensitivity and behavioral responses to the pheromone androstenone are not mediated by the vomeronasal organ in domestic pigs. Brain Behav. Evol..

[31] Groenen MA (2012). Analyses of pig genomes provide insight into porcine demography and evolution. Nature.

[32] Niimura Y, Nei M (2007). Extensive gains and losses of olfactory receptor genes in mammalian evolution. PLoS ONE.

[33] Moulton DG (1967). Olfaction in mammals. Am. Zool..

[34] Porter V (1993). Pigs: A Handbooks to the Breeds of the World.

[35] Jones, G. F. in *Genetic Aspects of Domestication, Common Breeds and Their Origin* (eds M. F. Rothschild & A. Ruvinsky) 17–50 (CAB International, 1998).

[36] Urbani G, Distrutti E, Biagioli M, Marchiano S, Fiorucci S (2022). How smell regulates metabolism: The role of ectopically expressed olfactory receptors in lipid and glucose homeostasis. J. Transl. Sci..

[37] Menashe I, Man O, Lancet D, Gilad Y (2002). Population differences in haplotype structure within a human olfactory receptor gene cluster. Hum. Mol. Genet..

[38] Gilad Y (2000). Dichotomy of single-nucleotide polymorphism haplotypes in olfactory receptor genes and pseudogenes. Nat. Genet..

[39] Kajiya K (2001). Molecular bases of odor discrimination: Reconstitution of olfactory receptors that recognize overlapping sets of odorants. J. Neurosci..

[40] Gilad Y, Bustamante CD, Lancet D, Paabo S (2003). Natural selection on the olfactory receptor gene family in humans and chimpanzees. Am. J. Hum. Genet..

[41] Dreger DL (2016). Whole-genome sequence, SNP chips and pedigree structure: building demographic profiles in domestic dog breeds to optimize genetic-trait mapping. Dis. Model Mech..

[42] Schiavo G (2020). Comparative evaluation of genomic inbreeding parameters in seven commercial and autochthonous pig breeds. Animal.

[43] Niimura Y (2012). Olfactory receptor multigene family in vertebrates: From the viewpoint of evolutionary genomics. Curr. Genomics.

[44] Celis-Giraldo CT (2021). A comparative analysis of SLA-DRB1 genetic diversity in Colombian (creoles and commercial line) and worldwide swine populations. Sci. Rep..

[45] Le MT (2020). SLA-1 genetic diversity in pigs: Extensive analysis of copy number variation, heterozygosity, expression, and breed specificity. Sci. Rep..

[46] Youk S (2022). Development of a high-resolution typing method for SLA-3, swine MHC class I antigen 3. Anim. Genet..

[47] Thong LM (2011). Systematic analysis of swine leukocyte antigen-DRB1 nucleotide polymorphisms using genomic DNA-based high-resolution genotyping and identification of new alleles. Tissue Antigens.

[48] Le MT (2012). Comprehensive and high-resolution typing of swine leukocyte antigen DQA from genomic DNA and determination of 25 new SLA class II haplotypes. Tissue Antigens.

[49] Park K (2010). Simple and comprehensive SLA-DQB1 genotyping using genomic PCR and direct sequencing. Tissue Antigens.

[50] Balakirev ES, Ayala FJ (2003). Pseudogenes: Are they “junk” or functional DNA?. Annu. Rev. Genet..

[51] Podlaha, O. & Zhang, J. *Pseudogenes and Their Evolution*. *eLS* (2010).

[52] Li WH, Gojobori T, Nei M (1981). Pseudogenes as a paradigm of neutral evolution. Nature.

[53] Hayden S (2010). Ecological adaptation determines functional mammalian olfactory subgenomes. Genome Res..

[54] Yohe LR, Fabbri M, Hanson M, Bhullar BS (2020). Olfactory receptor gene evolution is unusually rapid across Tetrapoda and outpaces chemosensory phenotypic change. Curr. Zool..

[55] Jeon H (2019). Copy number variation of PR-39 cathelicidin, and identification of PR-35, a natural variant of PR-39 with reduced mammalian cytotoxicity. Gene.

[56] Sambrook J, Russell DW (2001). Molecular Cloning: A Laboratory Manual.

[57] Ballester M, Castello A, Ramayo-Caldas Y, Folch JM (2013). A quantitative real-time PCR method using an X-linked gene for sex typing in pigs. Mol. Biotechnol..

[58] Ballester M, Castello A, Ibanez E, Sanchez A, Folch JM (2004). Real-time quantitative PCR-based system for determining transgene copy number in transgenic animals. BioTechniques.

[59] Wang J (2012). A genome-wide detection of copy number variations using SNP genotyping arrays in swine. BMC Genomics.

[60] Livak KJ, Schmittgen TD (2001). Analysis of relative gene expression data using real-time quantitative PCR and the 2(-Delta Delta C(T)) method. Methods.

[61] Smouse RPP, Peakall R (2012). GenAlEx 6.5: Genetic analysis in Excel. Population genetic software for teaching and research—An update. Bioinformatics.

[62] Excoffier L, Lischer HE (2010). Arlequin suite ver 3.5: A new series of programs to perform population genetics analyses under Linux and Windows. Mol. Ecol. Resour..

[63] R Core Team. R: A language and environment for statistical computing v. 4.1.2 (R Foundation for Statistical Computing, Vienna, Austria, 2021).

[64] Kumar S, Stecher G, Li M, Knyaz C, Tamura K (2018). MEGA X: Molecular evolutionary genetics analysis across computing platforms. Mol. Biol. Evol..

[65] Rozas J (2017). DnaSP 6: DNA sequence polymorphism analysis of large data sets. Mol. Biol. Evol..

