# Supplementary material for: Individual and population diversity of 20 representative olfactory receptor genes in pigs
Source: Sci Rep. 2023 Oct 31;13:18668. doi: 10.1038/s41598-023-45784-y (PMC10618239; doi:10.1038/s41598-023-45784-y)
Supplement: Supplementary file 1 — Supplementary Figures. [file 41598_2023_45784_MOESM1_ESM.docx]

Individual and population diversity of 20 representative olfactory receptor genes in pigs

Mingue Kang^1^, Byeongyong Ahn^1^, Seungyeon Youk^1^, Hyoim Jeon^1^, Nagasundarapandian Soundarajan^1^, Eun-Seok Cho^2^, Woncheoul Park^2^, and Chankyu Park^1*^

^1^ Department of Stem Cell and Regenerative Biotechnology, Konkuk University, Seoul 05029, Republic of Korea

^2^ National Institute of Animal Science, Rural Development Administration, Wanju 55365, Republic of Korea

*Corresponding Author

E-mail: [chankyu@konkuk.ac.kr](mailto:chankyu@konkuk.ac.kr) (CP)

**Running title:** Genetic diversity of pig olfactory receptor genes

**ORCID**

Chankyu Park: <https://orcid.org/0000-0003-4855-2210>


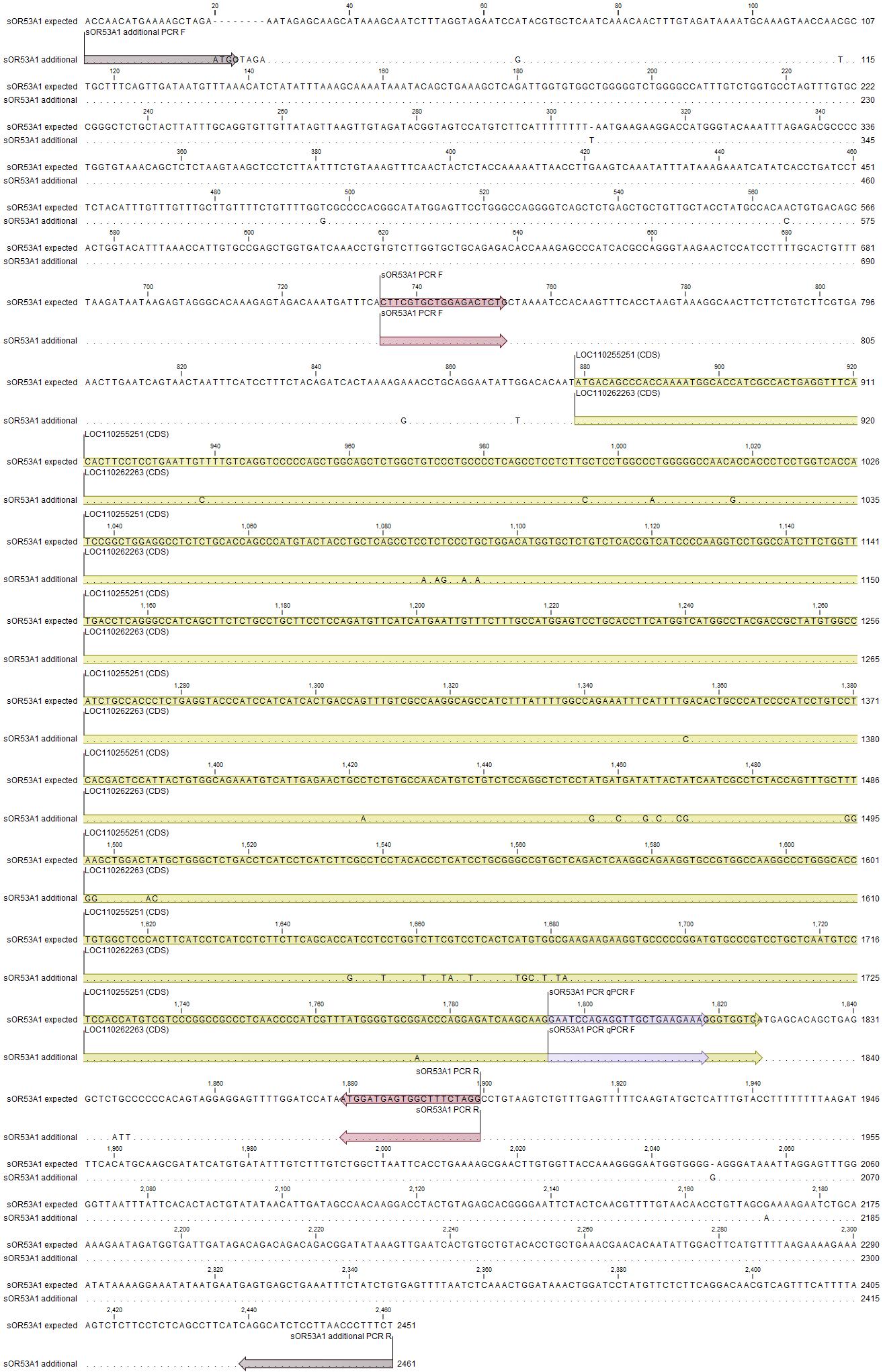
(A)


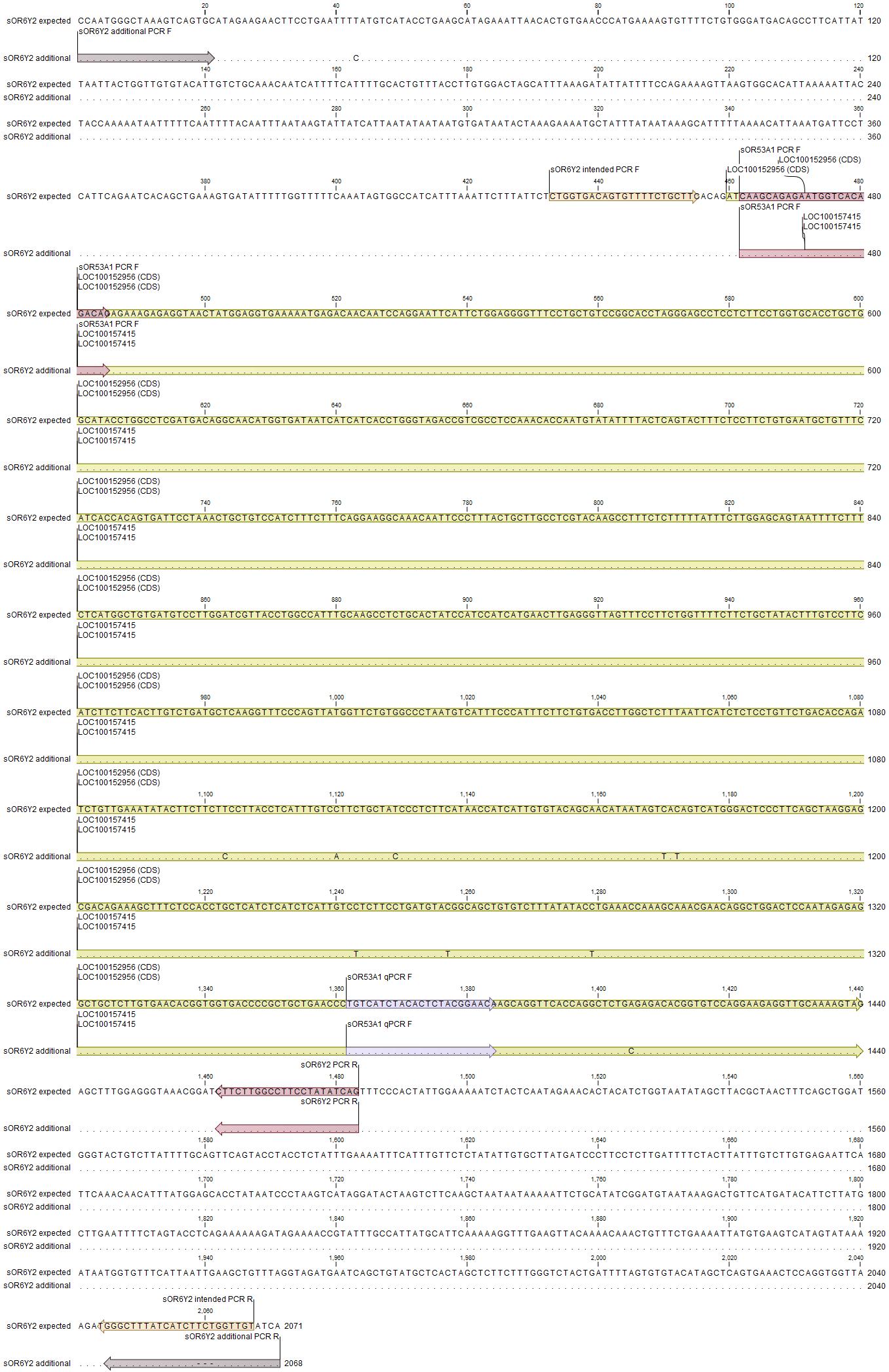
(B)

**S1 Fig. Positions of sequence-specific primers for *sOR53A1*- (A) and *sOR6Y2*-related (B) sequences.** Primer information for specific amplification of the expected sequences, according to the pig genome reference and the additionally detected sequence from OR typing in this study, are shown. Identical nucleotides are indicated by dots. The CDS regions are indicated with different colors.


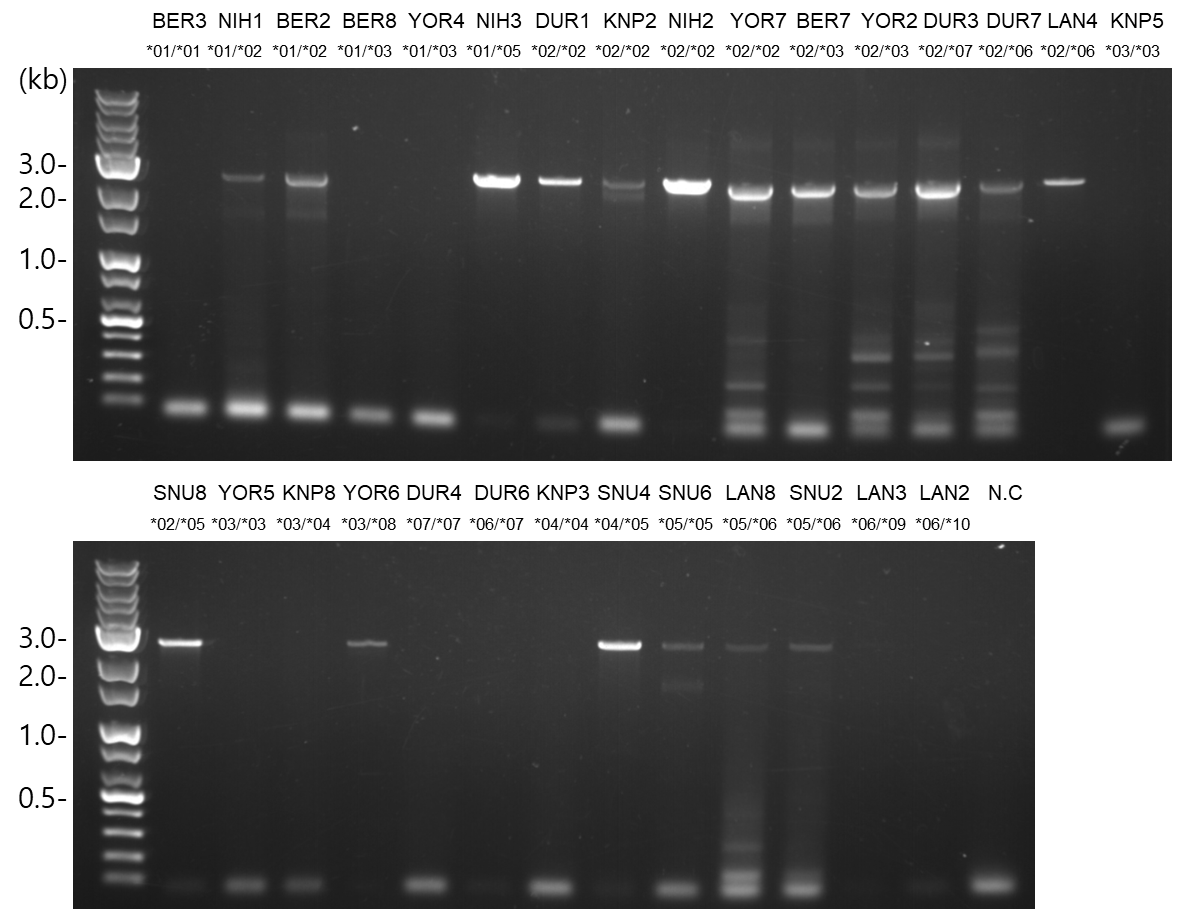
(A)


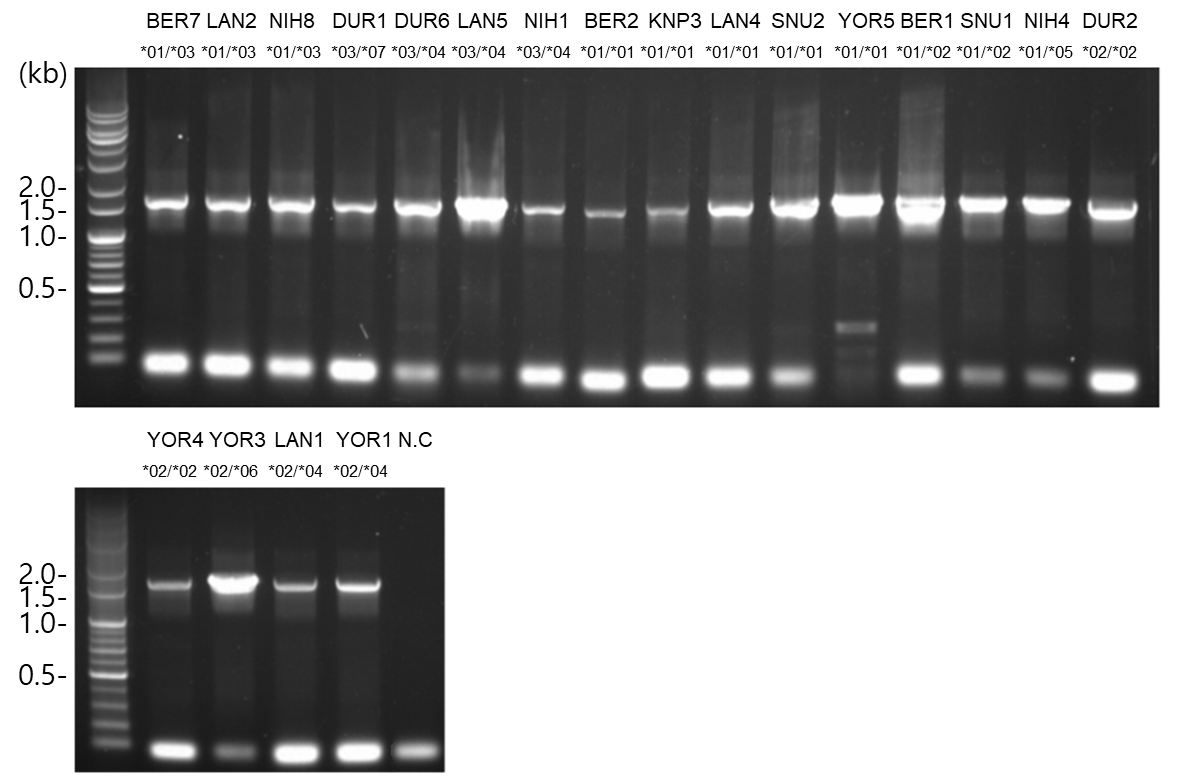
(B)

**S2 Fig. Results of sequence-specific typing for *sOR53A1* and *sOR6Y2* from 29 pigs.** (A) Amplification of the *sOR53A1*-related additional sequence (2,461 bp) using specific primers. (B) Amplification of *sOR6Y2* reference sequence of 1,635 bp using specific primers. Primer information is described in S1 Fig. Sample names, together with genotypes of the corresponding genes in our typing, are shown at the top of the gel. N.C, negative control. A DNA ladder is indicated on the left side of the gel.


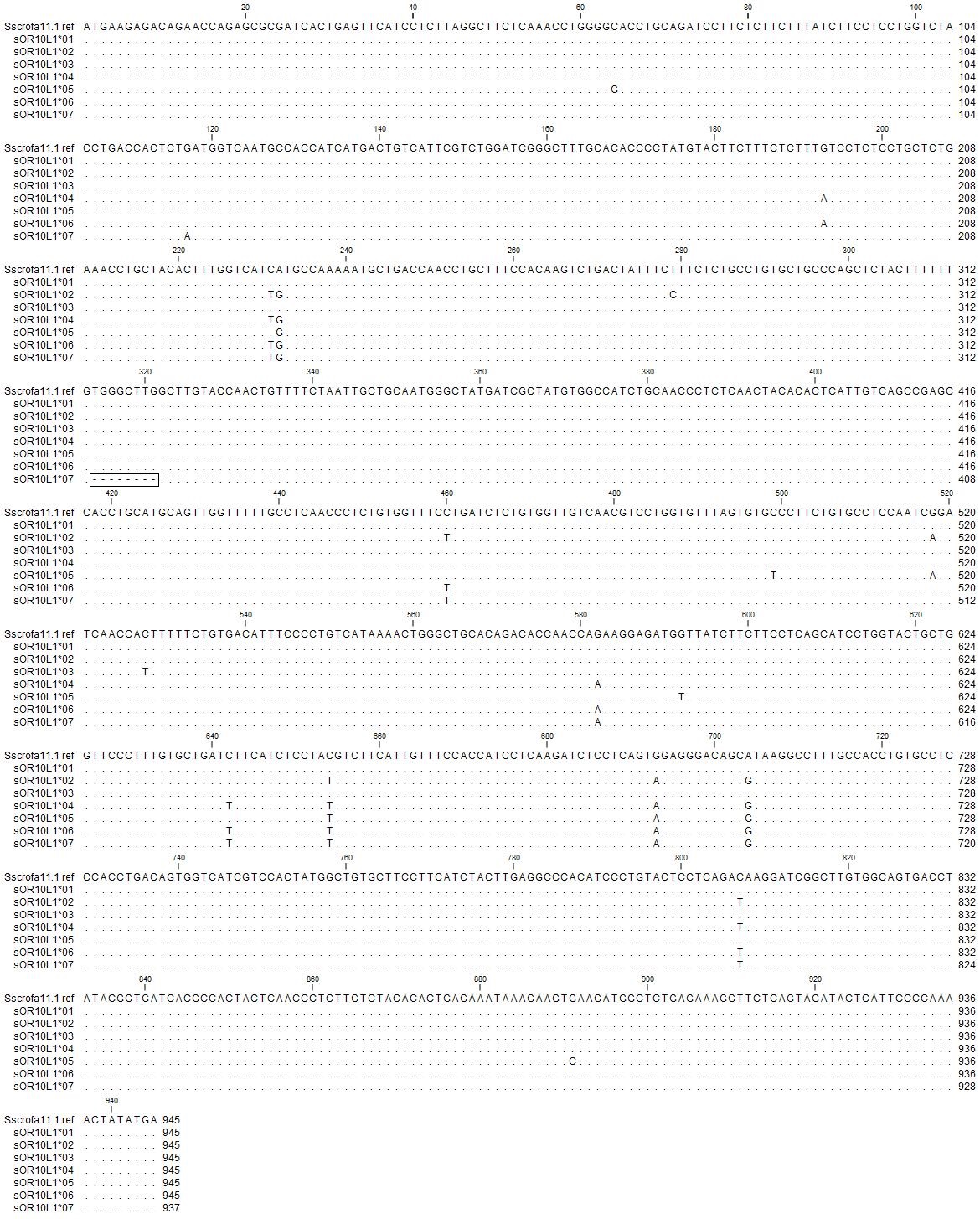
 **S3 Fig. Multiple sequence alignment of all identified *sOR10L1* alleles.** Identical nucleotides are represented by dots. The OR sequence of the pig genome assembly Sscrofa11.1 was used as a reference sequence. The indels on *sOR10L1*07* are marked with a square box.


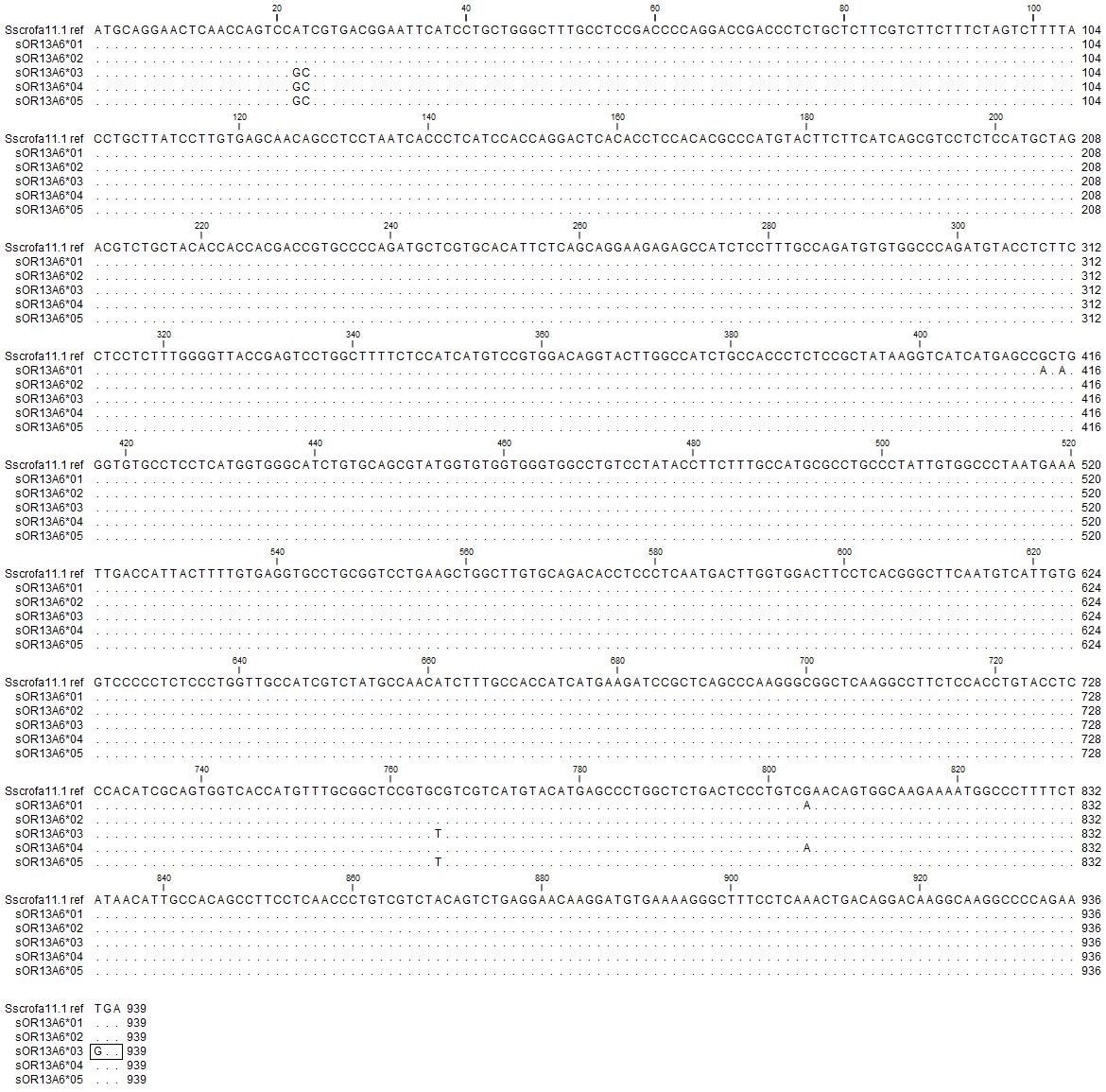
 **S4 Fig. Multiple sequence alignment of all identified *sOR13A6* alleles.** Identical nucleotides are represented by dots. The OR sequence of the pig genome assembly Sscrofa11.1 was used as a reference sequence. A nonsense mutation (TGA (stop) 🡪 GGA (Gly)) on *sOR13A6*03* is marked with a square box.


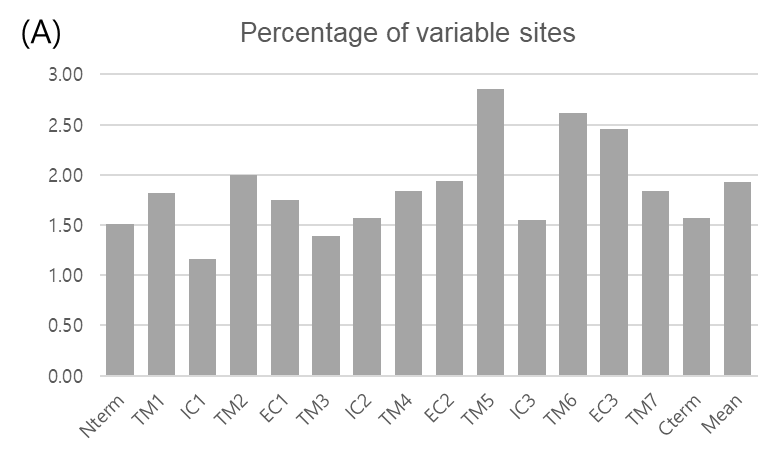

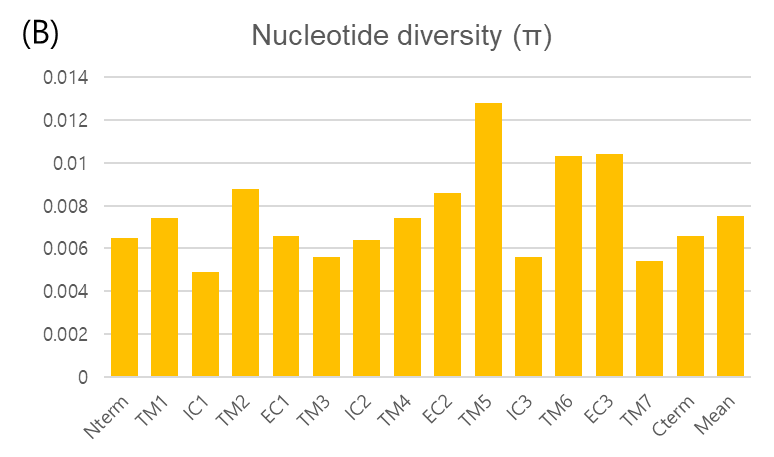

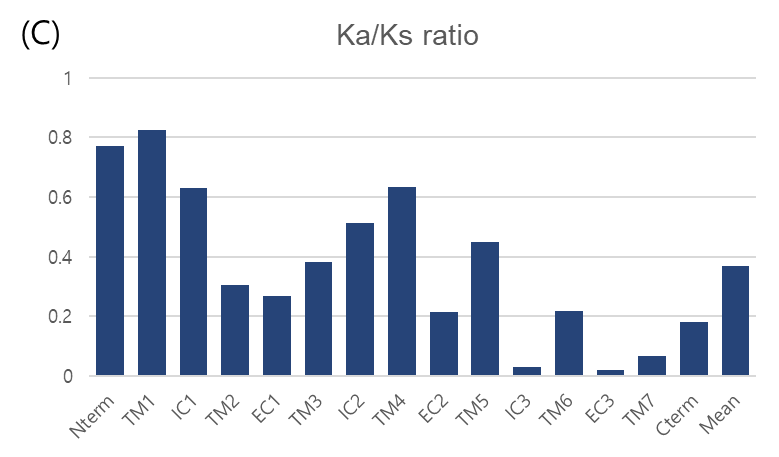


**S5 Fig. Comparison of the genetic indices of different OR structural domains in 20 pig OR genes.** Percentage of variable sites (A), nucleotide diversity (π) (B), and Ka/Ks ratio (C) for the 15 structural domains of 20 OR genes were plotted. Nterm, N-terminal domain; TM, transmembrane domain; IC, intracellular domain; EC, extracellular domain; Cterm, C-terminal domain.


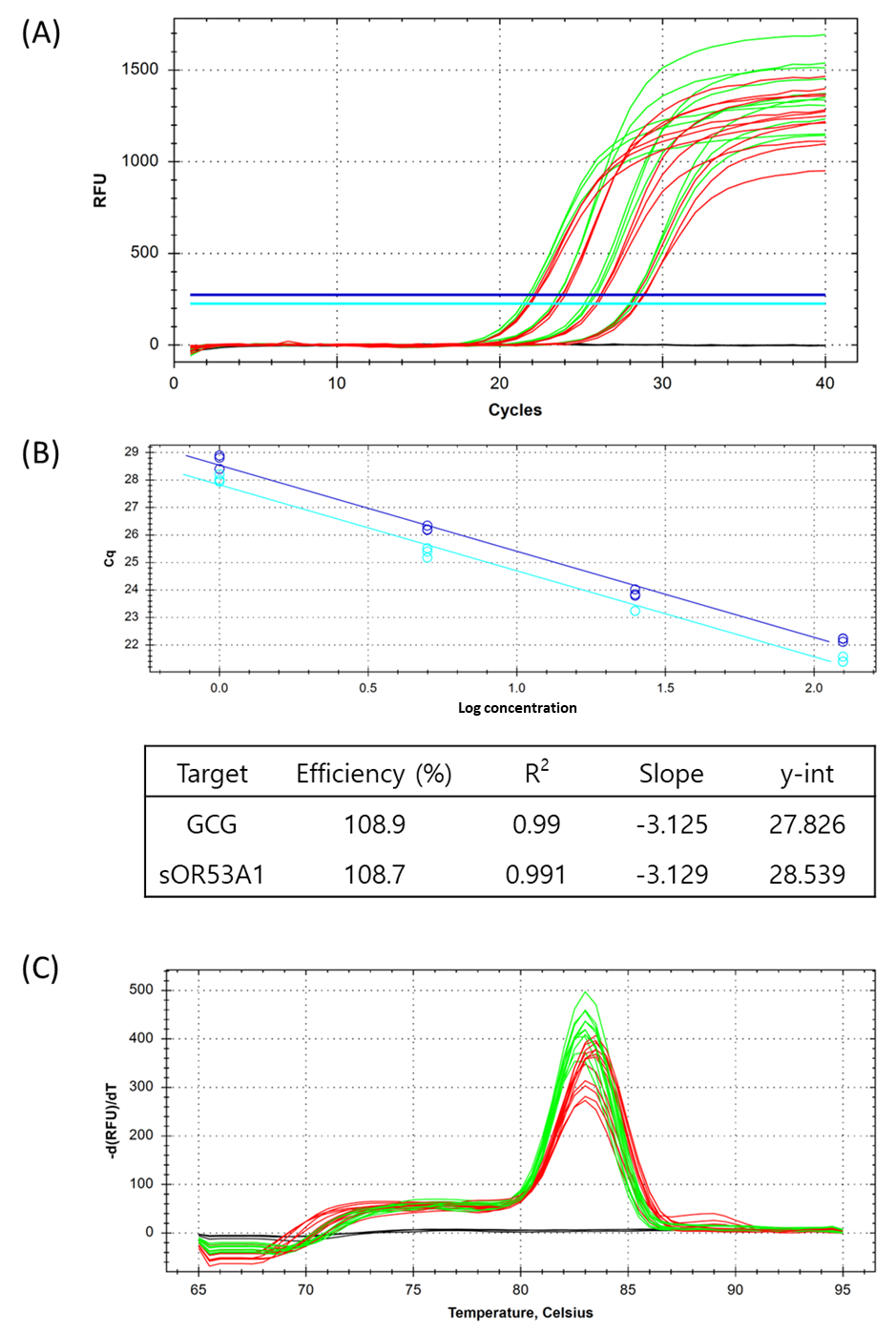


**S6 Fig. Evaluation of primer efficiency for *sOR53A1* and *GCG* qPCR.** The amplification plot (A), standard curve, qPCR efficiency (B), and melting curve (C) of each target were generated using 1, 5, 25, and 125 ng of pig genomic DNA from an individual (KNP3) with single-copy *sOR53A1*. The experiment was repeated thrice for each reaction. (A) Results for *GCG* and *sOR53A1* are shown in green and red, respectively. The negative controls are shown as black lines. To determine the cycle threshold (Cq), relative fluorescence units (RFU) of 225.6 and 274.1 were used for *GCG* (cyan) and *sOR53A1* (blue), respectively. (B) Standard curves for *GCG* (cyan) and *sOR53A1* (blue) are plotted according to the change in Cq with log concentration of genomic DNA. qPCR efficiencies were calculated from standard curves, and the values are presented in the plot. (C) Melting curves of all *GCG* and *sOR53A1* amplifications are plotted with green and red curves, respectively. Negative controls are shown as black lines. The melting temperature of each PCR run was based on the level of fluorescence reduction corresponding to −d(RFU)/dT.
